# Medical students’ perceptions towards distance e-Learning in gynecology ward during the COVID-19 pandemic

**DOI:** 10.1371/journal.pone.0283253

**Published:** 2023-03-24

**Authors:** Samaneh Rokhgireh, Babak Sabet, Shahla Chaichian, Farzaneh Derakhshan, Ladan Haghighi, Roya Derakhshan, Noushin Kohan

**Affiliations:** 1 Endometriosis Research Center, Iran University of Medical Sciences, Tehran, Iran; 2 Faculty of Medicine, Department of Surgery, Shahid Beheshti University of Medical Sciences, Smart University of Medical Sciences, Tehran, Iran; 3 Computer Science Department, Carnegie Mellon University, Pittsburgh, PA, United States of America; 4 Department of Medical Education, Smart University of Medical Sciences, Tehran, Iran; Pondicherry Institute of Medical Sciences, INDIA

## Abstract

**Introduction:**

The coronavirus disease 2019 (COVID-19) pandemic has caused serious public health problems and compromised the health of individuals and communities. This study aimed to evaluate a Distance e-Learning from the perspective of medical students in the Gynecology ward during the COVID-19 Pandemic.

**Material and methods:**

This cross-sectional study was conducted at the Iran University of Medical from the September 2020 to September 2021. The study sample included 130 medical students who participated in distance training courses in the gynecology ward during the COVID-19 pandemic. All medical students were included for the study. Medical students (externs and interns), who received Distance eLearning in the gynecology ward during the study, were included. The self-administered questionnaire was used in this study. Questionnaires was developed through literature review and consultation with gynecology and eLearning experts. Face and content validity was established by eight experts. Internal consistency was assessed with Cronbach’s alpha.

**Results:**

The questionnaire was sent to 170 medical students. Of the 130 respondents 65% were female and 35% were male. There were 57 (43.8%) externs and 73 (56.2) interns. Most students agreed that mobile devices increase their learning and home is the preferred place for participation in DE. Most students (66.9%) either strongly disagreed or disagreed that Distance e-Learning was an appropriate method for learning basic clinical skills.72.3% of respondents strongly disagreed or disagreed that Distance e-Learning provided them an opportunity to practice clinical skills effectively. Most medical students (69.3%) strongly agreed or agreed that Distance e-Learning created more opportunities to apply theoretical knowledge directly to medical practice.

**Discussion:**

The results of the online survey suggest that medical students have found both positive and negative aspects of clinical learning by DEL format in Gynecology ward.

## Introduction

The coronavirus disease 2019 (COVID-19) pandemic has caused serious public health problems and compromised the health of individuals and communities [1]. The spread of the COVID-19 virus across the world has affected the educational systems worldwide, including in Iran. The closure of educational institutions have created numerous challenges for medical students and teachers [2].

In response to the COVID-19 situation, the Association of American Medical Colleges made an announcement on March 17, 2020, and issued guidelines for medical schools to pause clinical rotations [3]. The National COVID-19 Administration in Iran also announced an emergency state [4]. Hence, all medical schools were forced to search for creative techniques to foster clinical medical education.

Distance e-Learning (DEL) emerged as the method of teaching to maintain the continuity of education during the pandemic [5]. DEL is defined as “using computer technology to deliver training, including technology-supported learning online, offline, or both” [6]. This shift was considered the best solution to clinical education problems in the era of COVID-19. The effectiveness of DEL amidst the COVID-19 crisis‏ has been investigated in several studies [5,7,8]. A survey by Al-Balas et al. showed that distance learning was the best solution to maintain clinical learning processes in emergency situations such as the COVID-19 pandemic [6].

In Iran’s medical education field, eLearning was not considered a new phenomenon in teaching medical students. The pandemic forced the use of virtual teaching and learning processes to complete the syllabus within the time frame. The Universities in Iran has implemented several technological tools to secure the remote clinical teaching process. Training sessions took place both for medical students and clinical teachers to learn how to interact via specific platforms, such as Adobe Connect, Skyroom, and Big Blue Button software. Nevertheless, the characterization of this format of teaching is perceived to be more difficult compared to face-to-face teaching for medical students [9,10].

In this regard, it is crucial to evaluate the perception of medical students on DEL approaches in the COVID-19 pandemic. This research focuses on the experience of DEL in clinical teaching at the Iran University of Medical Sciences from medical students’ viewpoints. This study aims to explore medical students’ perspectives on DEL in the Gynecology Ward during the COVID-19 pandemic.

## Material and methods

This cross-sectional study was conducted at the Iran University of Medical Sciences from September 2020 to September 2021. The study sample included 130 medical students, in the fifth and sixth year of Medicine who participated in distance training courses in the gynecology ward during the COVID-19 pandemic. All medical students were included for the study. A self-administered questionnaire was used in this study. Questionnaires were developed through literature review and consultation with gynecology and eLearning experts. Face and content validity was measured using qualitative methods. To measure face validity, 5 expert were selected and asked to check the questionnaire items respecting simplicity and clarity. The same experts are asked to examine the content validity of items with respect to relevance and representativeness. In this process, some items were added, deleted, and revised. Internal consistency was assessed with Cronbach’s alpha (0.83). The Thirty-item questionnaire consisted of six factors: (Demographic data, preferred eLearning tools and location for use, theoretical education, practical skills education, and instructor and infrastructure using a five-point Likert scale ("strongly agree," "agree," "no idea," "disagree," and "strongly disagree"). The research objectives were explained to the participants before the study. In addition, written informed consent was obtained. An online questionnaire was provided using Porsline software in the Persian language. Microsoft office excel software used for the data analysis. The result of the study was analyzed by using descriptive statistics such as frequency distribution and percentage. The link to the questionnaire was forwarded to the students by email. The Broadband internet connections were available to a much wider group of medical student who has participated in this study. Internet access for those who lack home broadband internet, were mobile access via their smartphones. Some medical students turn to public internet use (hospital or library) to participate in online class. Broadband internet connections were available to the instructors also (gynecology faculties). They broadcasted from their homes or the hospital.

Remote online classes were held synchronously via Adobe Connect, Skyroom, and Big Blue Button software and recordings were accessible afterwards. Online teaching was planned according to the educational calendar.

We used some strategies for engaging medical student during online classes such as using online group debate that promotes individual and group reflections in class, watching interactive videos, using a quiz during a synchronous learning activity, setting collaborative group tasks and opening student and teacher webcams.

The ethics committee approved this study at the Iran University of Medical Sciences (code: IR.IUMS.REC.1400.208).

## Results

The questionnaire was sent to 170 medical students. Of the 130 respondents 65% were female and 35% were male. There were 57 (43.8%) externs and 73 (56.2) interns. Preferred devices and locations for participation in Distance E-learning Courses is shown in Table 1. Table 2 shows the frequency distribution of respondents regarding medical students’ perceptions of DEL in the Gynecology ward during the Covid-19 Pandemic.

**Table 1:** Preferred eLearning tools and place for participation.

Preferred Place for participation in DEL				Preferred Applications for participation in DEL			
Dormitory	Educational department	Library	Home	PC	Laptop	Tablet	Mobile phone
11.9%	8%	7.7%	72.4%	1.1%	20.3%	10.1%	68.5%

**Table 2:** Frequency distribution of 130 respondents regarding to medical students’ perceptions towards DEL in gynecology ward during the Covid-19 pandemic.

Domains	Items	Strongly agree and agree %	Strongly disagree And disagree %	No Idea
**Clinical skills education**	Distance E-Learning was an appropriate method for learning basic clinical skills.	17	66.9	16–1
	Distance E-Learning provided me an opportunity to practice clinical skills effectively.	14.6	72.3	13.1
	It was possible to give feedback in demonstration of clinical skills.	19.2	63.1	17.7
	The virtual training covered most of the core clinical topics in the gynecology department.	26.2	58.5	15.3
	The assessment of clinical skill was effective in virtual format	20.8	61.5	17.7
**Theoretical skills education**	I have more opportunities to apply theoretical knowledge to medical practice.	69.3	20.7	10
	I have more opportunities to use theoretical topics and exchange theoretical knowledge.	57.7	25.4	16.9
	Attendance at virtual morning report sessions allowed a better experience for learning theoretical knowledge.	50	23	27
	Attendance at the virtual Journal club presentation allowed better experience for learning theoretical knowledge.	27.7	26.1	46.2
	Problem-solving and access to more resources were facilitated in the virtual environment.	54.6	24.6	20.8
	The quality of the virtual presentation of theoretical topics was not different from in-person classes.	44.6	43.1	12.3
	The theoretical topics were assessed effectively.	65.4	15.4	19.2
	The quality of virtual assessment was similar to face to face assessments.	59.2	23.1	17.7
	Generally, virtual education covered most of the practical topics required in the ward.	70.8	20	9.2
**Instructor competencies and infrastructure support**	Having a computer and a connection to the internet was enough to deliver the theoretical class.	23	35.4	41.6
	Having a computer and internet connection was enough to deliver clinical practice class.	26.1	53.8	20.1
	The teachers had enough skills and experience to teach theoretical subjects virtually.	73.8	13	13.2
	The teachers had enough skills and experience to teach clinical practice subjects virtually.	39.2	37.7	23.1
	The teachers provided an opportunity for discussion, teamwork and reflection.	64.6	25.4	10
	The virtual assessment of practical skills was desirable.	24.6	40	35.4
	There was proper planning for practical and theoretical training in virtual format.	51.5	28.5	20
	Virtual clinical training was an excellent alternative to conventional training.	50	38.4	11.6
	Virtual clinical training, provided more opportunities for direct education by the educator.	47.7	37.7	14.6

## Discussion

The results of the online survey suggest that medical students have found both positive and negative aspects of clinical learning by DEL format in Gynecology ward.

The results displayed in Table 1, are consistent with those of other studies and suggest that m-learning is an effective method for digital learning through the current and future crises, same as the COVID-19 pandemic. Therefore, the preparation for mobile learning would be important to manage future global threats [11]. This finding is in agreement with Bacolod’s (2022) findings which showed mobile learning as a crucial learning tool and agreed about its importance during pandemic despite some difficulties in implementation [12]. These findings further support the idea that m-learning is very helpful in improving the study gap during COVID-19 pandemic and education policymakers must incorporate mobile learning technology in educational systems [13]. Additionally, in a study by Ronnie et al. from the Philippines, 93% the students had a smartphone, and 83% had a laptop or computer.

The results displayed in Table 2, support the previous research that medical students had reported difficulties in clinical learning related to online teaching strategies during the pandemic [13]. A study by Dost et al. in 2020 showed that 75.99% of the medical students reported that online teaching had not positively replaced the clinical teaching that they experienced via bedside teaching [14]. This finding is also different from the result of the study by Hasan et al (2020) that 21.1% of medical students agreed that e-learning could be used effectively for clinical skill education [15]. A result of the study by Daffalla-Awadalla Gismalla (2021) showed that 64% of medical students perceived that online learning is the greatest strategy during COVID-19 lockdown [16]. In this regard, a study by Elzainy et al. in Saudi Arabia showed that 78% of students agreed or strongly agreed that e-learning had compensated for the suspension of in-person education due to the COVID-19 pandemic [17]. A result a study by Rafay et al. (2022) showed that 65% considered online education to be an unproductive teaching technique. Only 25% of the students required to be examined online however 34.5% were of the view that online oral examination was not good technique of examination [18].

Medical students were asked about their attitudes towards theoretical skills learning by DEL format. The findings of the current study are consistent with those of Tayem et al (2022) who found that 73.3% of medical students preferred distance learning for theoretical parts and students described that distance learning, improved interaction with teachers and peers. (45.6% and 48.9%, respectively). 60.1% of medical students were comfortable with online assessment [19]. In the study by Elzainy et al. (2020), 60% of medical students strongly agreed or agreed with the effectiveness of online assessments for determining their knowledge level, and 80% strongly agreed or agreed with online teaching of some theoretical courses [17].

Medical students were asked to report on their perceived level of instructor competencies and infrastructure support (See Table 2). In a study by Mortagy et al. (2022), many medical students (44.1%) said that their instructors were not well ready for online education. A significant percentage of medical students (45.2%) stated that having internet. difficulties such as internet connection problems and or internet speed [20]. In study of by Saurabh et al. (2021), about 62.7% of medical students had internet access. 67% of students were prepared to actively communicate with their peers and teachers electronically. 20.5% of medical students had linked online learning from home to conventional lectures [21].

However, with a small sample size, caution must be applied, as the findings might not be transferable to other contexts. Although the current study is a descriptive study that uses the perceptions of medical students in Iran University the findings must be inferred with caution.

## Conclusion

Medical students had different opinion about importance and application of the distance eLearning in clinical education. Some of them believed that this method was a good tool that helped them in their coverage of most of the practical topics required in the Gynecology ward and their teachers had enough skills and experience to teach theoretical subjects virtually and this method provided an opportunity for discussion, teamwork and reflection for them. On the one hand, participants were not believed Distance E-Learning was an appropriate method for learning basic skills and practicing clinical skills effectively. Faculty development of medical teachers in online teaching methods also need to be considered. The results of this study have implications for policy and practice with regard to distance eLearning clinical education training in Iran.

## Supporting information

S1 File (SAV)

## References

[1] Varshney M, Parel JT, Raizada N, Sarin SK. Initial psychological impact of COVID-19 and its correlates in Indian Community: An online (FEEL-COVID) survey. PLoS One. 2020;15(5):e0233874. doi: 10.1371/journal.pone.0233874 32470088 PMC7259495

[2] Adedoyin OB, Soykan E. Covid-19 pandemic and online learning: the challenges and opportunities. Interact Learn Environ. 2020:1–13.

[3] Schuiteman S, Ibrahim NI, Hammoud A, Kruger L, Mangrulkar RS, Daniel M. The role of medical student government in responding to COVID-19. Acad Med. 2020, Jun 9. 10.1097/ACM.0000000000003542 PMC7302065 32520750

[4] Raoofi A, Takian A, Sari AA, Olyaeemanesh A, Haghighi H, Aarabi M. COVID-19 pandemic and comparative health policy learning in Iran. Arch Iran Med (AIM). 2020;23(4).220–234. doi: 10.34172/aim.2020.02 32271594

[5] Ennam A. Assessing Covid-19 pandemic-forced transitioning to distance e-learning in Moroccan universities: an empirical, analytical critical study of implementality and achievability. J North Afr Stud. 2021:1–25.

[6] Al-Balas M, Al-Balas HI, Jaber HM, Obeidat K, Al-Balas H, Aborajooh EA, et al. Distance learning in clinical medical education amid COVID-19 pandemic in Jordan: current situation, challenges, and perspectives. BMC Med Educ. 2020;20(1):1–7. 10.1186/s12909-020-02257-4 PMC7530879 33008392

[7] Rajab MH, Gazal AM, Alkattan K. Challenges to online medical education during the COVID-19 pandemic. Cureus. 2020 Jul 2;12(7). doi: 10.7759/cureus.8966 32766008 PMC7398724

[8] Sasidharan S, Dhillon H. Undergraduates medical education in the time of COVID-19: Lessons learnt from Democratic Republic of the Congo. N Z Med J. 2021(32):55–7.33767487

[9] Taghrir MH, Borazjani R, Shiraly R. COVID-19 and Iranian medical students; a survey on their related-knowledge, preventive behaviors and risk perception. Arch Iran Med (AIM). 2020;23(4):249–54. 10.34172/aim.2020.06 32271598

[10] Pourghaznein T, Salati S, Jamali J, Rangani F, Khazaei E. Study of behaviors and psychological indicators in Iranian medical students during the COVID-19 pandemic self-quarantine. J Health Lit. 2021;6(1):61–71.

[11] Al-Ajlan A, Zedan H, editors. The extension of web services architecture to meet the technical requirements of virtual learning environments (Moodle). 2008 International Conference on Computer Engineering & Systems; 2008: IEEE.

[12] Bacolod D. Mobile Learning as a Solution for Restricted Learning during the COVID-19 Pandemic. J Digit Educ Technol. 2022;2.

[13] Biswas B, Roy SK, Roy F. Students perception of mobile learning during COVID-19 in Bangladesh: university student perspective. 2020.

[14] Dost S, Hossain A, Shehab M, Abdelwahed A, Al-Nusair L. Perceptions of medical students towards online teaching during the COVID-19 pandemic: a national cross-sectional survey of 2721 UK medical students. BMJ open. 2020;10(11):e042378. doi: 10.1136/bmjopen-2020-042378 33154063 PMC7646323

[15] Alsoufi A, Alsuyihili A, Msherghi A, Elhadi A, Atiyah H, Ashini A, et al. Impact of the COVID-19 pandemic on medical education: Medical students’ knowledge, attitudes, and practices regarding electronic learning. PLoS One. 2020;15(11):e0242905. doi: 10.1371/journal.pone.0242905 33237962 PMC7688124

[16] Gismalla MD-A, Mohamed MS, Ibrahim OSO, Elhassan MMA, Mohamed MN. Medical students’ perception towards E-learning during COVID 19 pandemic in a high burden developing country. BMC Med Educ. 2021;21(1):1–7. 34246254 10.1186/s12909-021-02811-8 PMC8271314

[17] Elzainy A, El Sadik A, Al Abdulmonem W. Experience of e-learning and online assessment during the COVID-19 pandemic at the College of Medicine, Qassim University. JTUMED. 2020;15(6):456–62. doi: 10.1016/j.jtumed.2020.09.005 33106752 PMC7578775

[18] Rafay A, Hiba H-T, Jamil M, Arshad A, Hussain A, Malik AA, et al. Effect of COVID-19 on Medical Education and Examination: A Cross-Sectional Survey across Medical Colleges of Pakistan. EJMED. 2022;4(1):90–2.

[19] Tayem YI, Almarabheh AJ, Hamza EA, Deifalla A. Perceptions of Medical Students on Distance Learning During the COVID-19 Pandemic: A Cross-Sectional Study from Bahrain. Adv medical educ pract. 2022;13:345. doi: 10.2147/AMEP.S357335 35478974 PMC9037435

[20] Mortagy M, Abdelhameed A, Sexton P, Olken M, Hegazy MT, Gawad MA, et al. Online medical education in Egypt during the COVID-19 pandemic: a nationwide assessment of medical students’ usage and perceptions. BMC Med. 2022;22(1):1–13. doi: 10.1186/s12909-022-03249-2 35354406 PMC8966850

[21] Saurabh MK, Patel T, Bhabhor P, Patel P, Kumar S. Students’ perception on online teaching and learning during COVID-19 pandemic in medical education. Maedica. 2021;16(3):439. doi: 10.26574/maedica.2021.16.3.439 34925600 PMC8643544

